# Presenting your structures: the *CCP*4*mg* molecular-graphics software

**DOI:** 10.1107/S0907444911007281

**Published:** 2011-03-18

**Authors:** S. McNicholas, E. Potterton, K. S. Wilson, M. E. M. Noble

**Affiliations:** a York Structural Biology Laboratory, Department of Chemistry, University of York, Heslington, York YO10 5DD, England; b Department of Biochemistry, University of Oxford, South Parks Road, Oxford OX1 3QU, England

**Keywords:** *CCP*4*mg*, molecular graphics, movies, crystallographic software

## Abstract

*CCP*4*mg* is a molecular-graphics program that is designed to give rapid access to both straightforward and complex static and dynamic representations of macromolecular structures. It has recently been updated with a new interface that provides more sophisticated atom-selection options and a wizard to facilitate the generation of complex scenes. These scenes may contain a mixture of coordinate-derived and abstract graphical objects, including text objects, arbitrary vectors, geometric objects and imported images, which can enhance a picture and eliminate the need for subsequent editing. Scene descriptions can be saved to file and transferred to other molecules. Here, the substantially enhanced version 2 of the program, with a new underlying GUI toolkit, is described. A built-in rendering module produces publication-quality images.

## Abstract 2

The *CCP*4 molecular-graphics program now uses the Qt framework to provide a modern look and feel. There are many new features including rendering for publication-quality images and sequence alignment.

## 1. Introduction

Interactive molecular-graphics programs are central to the work performed by macromolecular crystallographers. *CCP*4*mg* has been developed as an easy-to-use yet flexible system for displaying the structure and structure-based properties of macromolecules (Potterton *et al.*, 2002 ▶, 2004 ▶). It is not the aim of this paper to provide a comprehensive review of the field of macromolecular graphics, but rather to describe the main features of *CCP*4*mg*. For the history of molecular graphics and the development of the many software packages, readers are directed to the excellent review by Lesk *et al.* (2008 ▶). However, a brief survey of other molecular graphics helps to illustrate their broad utility in different areas of biomolecular science.

*CCP*4*mg* builds upon the ideas for representing macromolecular structures encoded in programs such as *MolScript* (Kraulis, 1991 ▶) and its extended variant *BobScript* (Esnouf, 1999*a* ▶,*b* ▶), which both use the renderer *Raster*3*D* (Merritt & Bacon, 1997 ▶) to produce high-quality output. Further inspiration for *CCP*4*mg* is drawn from programs such as *RIBBONS* (Carson, 1997 ▶), *SPOCK* (Christopher *et al.*, 1996 ▶), *DINO* (http://www.dino3d.org/) and *INSIGHT* (Dayringer *et al.*, 1986 ▶), all of which have contributed many ideas to how large structures should be visualized. *MIDAS* (Ferrin *et al.*, 1988 ▶) has been a pioneering drug-design and molecular-viewing program and its successor *Chimera* (Petterson *et al.*, 2004 ▶) remains one of the most popular and powerful programs in the field. *VMD* (Humphrey *et al.*, 1996 ▶) was created to visualize molecular dynamics and has developed as an extremely capable general display package for large structures. *Swiss-PdbViewer* (Guex & Peitsch, 1997 ▶) is a molecular-graphics program designed to work with the *SWISS-MODEL* server for comparative protein modelling. *RasMol* (Sayle & Milner-White, 1995 ▶; Bernstein, 2000 ▶) employs efficient com­putational methods to achieve excellent performance and its command language was adopted by the popular viewer *Jmol* (D. Gezelter; http://www.jmol.org). *Chime* (created by MDL Systems, now part of Symyx; http://www.symyx.com/downloads/downloadable/) and *Protein Explorer* (Martz, 2002 ▶) were developed as variants of *RasMol* to perform interactive molecular graphics within a web browser. *AstexViewer* (Hartshorn, 2002 ▶) is another important example of a web-browser-based viewer. *Cn*3*D* (http://www.ncbi.nlm.nih.gov/Structure/CN3D/cn3d.shtml) is particularly well suited to the display of structural alignments of multiple proteins; it works with the NCBI’s MMDB and VAST databases of structures and alignments but not directly with PDB-format files. The SGC’s *iSee* (Abagyan *et al.*, 2006 ▶) program built upon Molsoft’s *ICM-Browser* technology provides a means of creating representations of chemical structure using a tight coupling of interactive molecular graphics, hypertext, tables *etc*. *PyMOL* (DeLano, 2002 ▶) is one of the most widely used of the current generation of molecular-graphics systems, but there are many others including *Qmol* (Gans & Shalloway, 2001 ▶), *YASARA* (http://www.yasara.org) and *CheVi* (http://www.simbiosys.ca/chevi/index.html).

The main aim of the *CCP*4*mg* project is to make available a tool for the visualization of the static and dynamic features of macromolecular structures. The program provides tools for simply and rapidly creating complex single scenes and movies. Such tools include the automatic generation of multiple drawing styles to emphasize important structural information, the highlighting of equivalent structural features of different models by the use of structure-based and sequence-based alignment and drawing molecular surfaces over a whole structure or selected regions to focus on details of molecular-recognition sites. The prediction and visualization of quaternary structure and molecular interfaces is achieved through the use of *PISA* (Krissinel & Henrick, 2005 ▶, 2007 ▶; Krissinel, 2010 ▶). Electron density can be drawn in many styles and over selected subsets of atoms to highlight areas of interest. Other features include the drawing of textual labels and geometric shapes and the overlaying of two-dimensional images from files to further enhance scenes. Care has been taken to make the creation of movies in which the view is rotated, zoomed, panned *etc*. to show various aspects of the model as intuitive as possible. Moreover, movies can be generated from a series of coordinate files in order to highlight changes in molecular structure. *CCP*4*mg* has a built-in rendering module (the open-source RenderMan-compliant *Pixie* engine; http://www.renderpixie.com/) to produce high-quality images for publication; the renderer may also be used to create the individual frames of a movie.

*CCP*4*mg* is free software (LGPL; http://www.gnu.org/licenses/lgpl.html) and can be downloaded from the website http://www.ccp4.ac.uk/MG/, where detailed documentation is also provided. In addition, a set of tutorials providing a simple guide for the new user is available at http://www.ccp4.ac.uk/MG/ccp4mg_help/tutorial/introduction.html.

## 2. Picture creation

### 2.1. Atomic model representation

*CCP*4*mg* can draw molecules in most commonly used styles, including ball and stick, simple lines, cylinders, space-filling spheres, ribbon cartoons for secondary structure and molecular surfaces. Other properties, for example hydrogen bonds, contacts and user-defined inter­atomic vectors, are depicted as independently defined objects. Solid representations such as surface and ribbons can be transparent, with user control of the extent of transparency.

The user may colour the atoms by atom type, residue type, chain, position in chain or molecule, various physical properties or by flexible customized selections. Structures can be coloured by secondary-structure type or solvent accessibility; these properties are calculated within the program.

Many different representations of many different selections of molecules can be displayed simultaneously to create complex scenes. The selections are not named in the same way as in, for example, *PyMOL* (DeLano, 2002 ▶), but may be saved and later restored or applied to other models. There are three levels of selection.

#### 2.1.1. Simple atom selections

**Figure 1:**
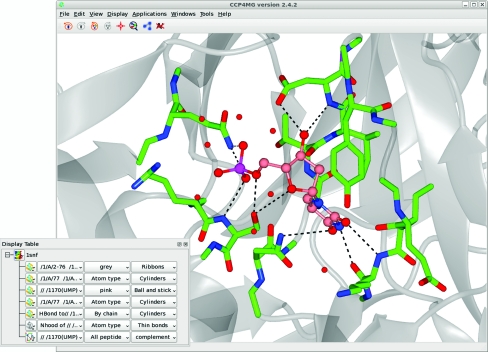
The *CCP*4*mg* main window and display table. Scene automatically created with Picture wizard style ‘ligand binding site: site and broken ribbons’ for PDB entry 1snf (Chan *et al.*, 2004 ▶).

The *CCP*4*mg* interface consists of two windows (Fig. 1 ▶). The main window contains the graphical display and a menu and toolbar for performing the most common tasks, such as file input and output.

A second window provides the display table, which contains information about all the currently loaded data items such as PDB files and electron-density maps. For each data item there is a menu corresponding to the various representations derived from that item. Each display object in the table has three menu buttons controlling its key properties. For the molecular-display objects these properties are (i) the subset of atoms used, (ii) the colour scheme applied and (iii) the drawing style that has been chosen. Display objects can be easily added or deleted or the selection and the colour and drawing style can be edited to build scenes.

#### 2.1.2. Advanced selection options

The next level in sophistication is the ‘Selection browser’ widget which provides more powerful tools for editing complex definitions of subsets of atoms from the PDB files that have been loaded. Firstly, there is a tree pane, which allows the user to see and select from the molecular structure in terms of a tree of model components (peptide, nucleic acid, monomers, solvent), chains, residues and atoms. Secondly, additional widgets allow selection by criteria such as contact distance and atom and residue properties. As an example, the user can select all the amino acids in chain *A* and then use the neighbourhood pane to specify that only residues within 6 Å are required and finally use the residue-types pane to specify acidic residues.

Internal to *CCP*4*mg* all selections are maintained as a string in a selection language; the user input to the various GUIs is converted to this selection string. The selection browser has a pane displaying the current selection command that the user can edit. Expert users may prefer the speed and flexibility of editing the selection string compared with changing the values of menus or checkboxes.

Complex scenes are created by the use of multiple representations with different atom selections and drawing styles. For example, one can draw some amino-acid residues as cylinders and the rest as a ribbon, all ligands as cylinders, metal atoms as spheres and solvent, sulfates *etc*. as ball and stick. The scene may be enhanced by the addition of a particular set of hydrogen bonds, such as those in the vicinity of a binding site and by a molecular surface over some or all atoms.

#### 2.1.3. The Picture wizard: automated picture styles

**Figure 2:**
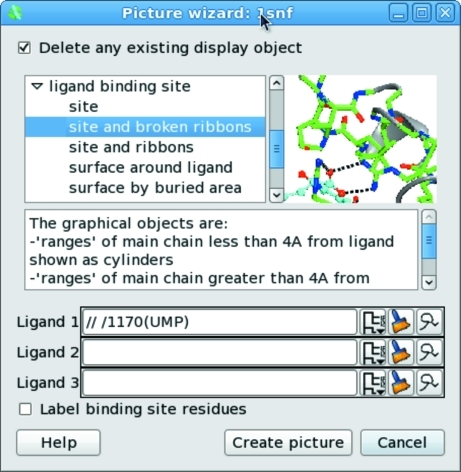
The Picture wizard. The selected style automatically created the scene shown in Fig. 1 ▶.

The ‘Picture wizard’ enables the automatic creation of complex sets of selections and styles without user intervention. The wizard styles have been developed based on user input to generate informative representations relevant to the most common usages of molecular graphics. The view created may subsequently be fine-tuned by using the Selection browser and controlling drawing-style preferences. The Picture wizard is available in the file browser when the molecule is loaded and can subsequently be accessed from the display table while using the program (Fig. 2 ▶). The available picture styles are grouped into general schemes that include ‘bonds’, ‘ribbons’, ‘nucleic acid’, ‘ligand-binding site’, ‘interfaces’ and ‘surfaces’. A small cartoon illustrates each picture style. The power of the wizard comes from its automatic analysis of the composition of the structure so that protein, nucleic acid, ligands, binding sites, interfaces and solvent are identified heuristically and automatically and are displayed in an appropriate style. This system is not as automated as the maximally informative initial views of, for example, *Chimera* (Petterson *et al.*, 2004 ▶) and *iSee* (Abagyan *et al.*, 2006 ▶), but may offer some an easier route into subsequent optimization.

### 2.2. Electron density

**Figure 3:**
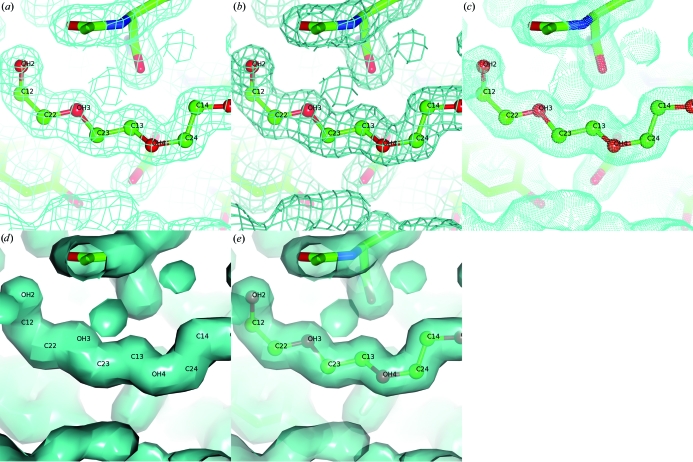
The electron-density styles possible in *CCP*4*mg*: (*a*) chickenwire, (*b*) chickenwire cylinders, (*c*) dots, (*d*) solid and (*e*) solid with transparency.

Electron-density maps are displayed as isosurfaces with line chickenwire, cylinder chickenwire or solid surface styles; a ‘multi-chickenwire’ map display style has recently been added that draws multiple contour values simultaneously to create an enhanced solid appearance (Fig. 3 ▶). The map is represented internally as a ‘continuous’ crystal: the centre of the contoured volume adapts to always correspond to the centre of the current view. Molecules related to the PDB coordinates by crystallographic symmetry are generated on the fly by application of the unit-cell and symmetry operators of the map. The map may be clipped to a selected set of atoms to highlight features of particular interest within the structure.

Much of the code to draw solid surface representations of electron density was donated by the *Coot* model-building project (Emsley & Cowtan, 2004 ▶; Emsley *et al.*, 2010 ▶).

### 2.3. Additional graphical objects: text annotations, overlays and vectors

**Figure 4:**
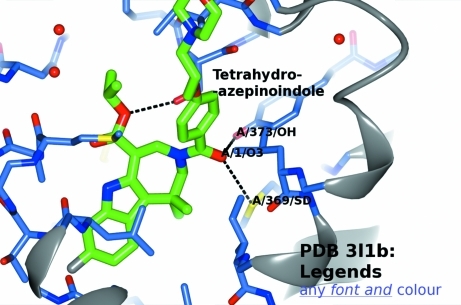
The text-display capabilities of *CCP*4*mg*. Three atoms are labelled with the default format: chain/residue number/atom name. The ligand has an annotation attached. All these rotate as the view is changed. In contrast, the ‘Legend’ is in a static user-defined screen position.

User-defined text annotations, which move with the structure as the view changes, can be attached to user-specified atoms in much the same way as the ‘Annotation’ feature in *Cn*3*D* (http://www.ncbi.nlm.nih.gov/Structure/CN3D/cn3d.shtml). In addition, a static text ‘Legend’ (any system font, bold, underlined or italic, with subscripts and superscripts and a choice of colour) can be added at a defined two-dimensional screen coordinate (Fig. 4 ▶). The facility for high-quality text minimizes the need to annotate images outside *CCP*4*mg*. Coupled with the facility for saving and restoring scenes, this makes editing images straightforward.

Images can be overlayed on a scene in any common bitmap format or as a scalable vector graphic (SVG). In ‘Geometry’ mode clicking on atoms shows distances, angles and torsion angles. The picture created can be enhanced by the addition of ‘vectors’ which connect either a user-specified pair of atoms or user-specified Cartesian coordinates with a line or arrow and an optional text label.

A recent feature allows the addition of arbitrary geometric primitives to the scene (polygons, lines, cylinders *etc*.) and text to enhance the information content of the structures.

## 3. Analysis tools: surfaces, structural and sequence alignments

### 3.1. Surfaces

Molecular surfaces (Lee & Richards, 1971 ▶; Connolly, 1983 ▶) coloured by various physical properties, including electrostatic potential, are displayed using the *CXXSurface* toolkit (Gruber *et al.*, 2007 ▶). These authors demonstrated the visualization of sequence conservation and hydrophobicity as examples of the importance of molecular surfaces in understanding the structure of biomolecules.

The electrostatic potential is calculated by solving the Poisson–Boltzmann equation, 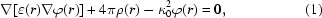
where ∊(*r*) is the relative permittivity at *r*, ρ(*r*) is the charge density arising from diffusable charges at *r* and κ_0_ is the Debye–Hückel screening parameter. Gruber and coworkers implemented an iterative finite-difference approach to solving the problem, which uses a rapid FFT-based method to determine the protein interior; the charge distribution is smoothed within the initial potential map. The values of the potential at convergence are allocated to each of the vertices of the graphical triangles that define the molecular surface. *CCP*4*mg* displays surfaces in a range of styles: a solid surface with optional transparency, a mesh representation or as dots. Surfaces over complete models and subsets of atoms are possible.

### 3.2. Structure alignment

*CCP*4*mg* provides three methods for structure superposition: the secondary-structure matching (*SSM*) algorithm (Krissinel & Hendrick, 2004 ▶), close-residue matching and user-defined matching. The appropriate superposition transformation matrices can be applied to any electron-density maps associated with the superposed models.

#### 3.2.1. *SSM*

*SSM* matches equivalent secondary-structure elements (SSEs) in pairs of structures. The equivalencies are determined to a first approximation by matching graphs built on a vectorial representation of the secondary-structure elements (Artymiuk *et al.*, 1989 ▶). Equivalent SSEs are those that have a close relative orientation and separation in distance in both structures. This gives an approximate superposition that is used to provide an initial base for matching individual C^α^ atoms. The match is then optimized using a function that balances the r.m.s.d. and the number of matched C^α^ pairs. *SSM* is able to superpose both closely similar structures and those with more distant relations. *SSM* has been incorporated into *CCP*4*mg*, thus allowing the user to select the target molecules for multiple structure alignments and to specify the regions of the structures to be superposed.

#### 3.2.2. Close-residue matching

This enables locally optimized superposition after a global superposition by *SSM*. For example, the user can optimize the superposition of the residues forming a particular ligand-binding site.

#### 3.2.3. User-defined matches

This allows the user to specify equivalent individual residues or atoms by hand. There is a tool to automatically find equivalent atoms in similar residues or ligands. This is particularly useful when trying to overlay the relevant parts of non-identical ligands.

### 3.3. Sequence viewer

**Figure 5:**
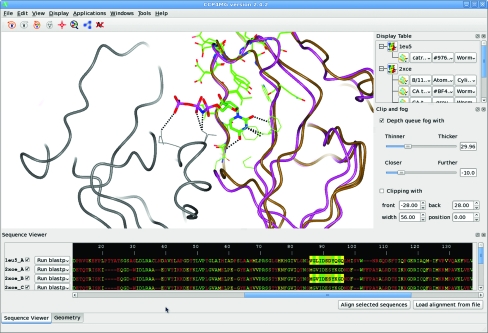
Structure and sequence alignment. Chain *A* of PDB entry 1eu5 (brown; González *et al.*, 2001 ▶) superimposed on chain *B* of 2xce (pink; García-Nafría *et al.*, 2010 ▶) using *SSM*. The nucleotide analogue dUpNpp bound to 2xce is drawn as cylinders. The sequences, aligned using *MUSCLE*, are displayed in the ‘Sequence viewer’ window coloured by conservation. The residues highlighted in the sequence viewer appear as stick representations in the graphics window. This figure also demonstrates the new single-window appearance of *CCP*4*mg*, with the Sequence viewer, Display Table and Clip and Fog widgets docked in the main window.

Recent releases contain a protein-sequence viewer in which the user can highlight sections of the structure (Fig. 5 ▶). This can be performed with multiple sequences and the development of more advanced alignment tools is in hand which will provide more extensive functionality. For example, recent builds include the ability to load sequences and alignments in many formats, including *Clustal*, *FASTA*, *GCG*/*MSF* and *PIR*, and to generate a sequence alignment using the *MUSCLE* tool (Edgar, 2004 ▶). Searching of databases for similar sequences to download and display any corresponding structures is also possible using the EBI/NCBI *BLAST* service (Johnson *et al.*, 2008 ▶; McWilliam *et al.*, 2009 ▶). The sequence viewer is a work in progress and does not yet provide the extensive functionalities of, for example, *Multialign Viewer* in *Chimera* (Petterson *et al.*, 2004 ▶).

### 3.4. *PISA*

*PISA* (*Protein Interfaces, Surfaces and Assemblies* service at the European Bioinformatics Institute; http://www.ebi.ac.uk/msd-srv/prot_int/pistart.html; Krissinel & Henrick, 2005 ▶, 2007 ▶; Krissinel, 2010 ▶) is a tool for the exploration of macromolecular (protein, DNA/RNA and ligand) interfaces, the prediction of probable quaternary structures (assemblies) and database searches for structurally similar interfaces and assemblies, as well as searches on various assembly and PDB-entry parameters.

*PISA* identifies the chemical monomers and the interfaces between them, including those generated by applying symmetry to the input coordinates. By evaluating the strength of interaction between the neighbouring monomers in a crystal, *PISA* predicts the most stable multimer likely to be the functional biological unit.

**Figure 6:**
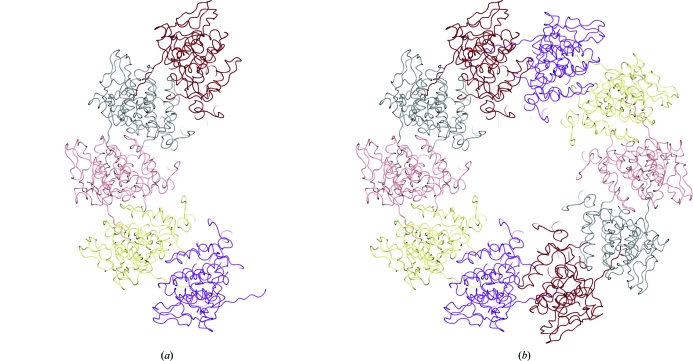
Assembly prediction by *PISA*. (*a*) The original model (PDB entry 3hhz; Green & Luo, 2009 ▶). (*b*) The dimer predicted by *PISA*.

*PISA* is available as an EBI web service and also as a standalone program which has been incorporated into *CCP*4*mg* to enable molecular-graphics visualization of the results. The monomers present are listed and the user may click on them to create display objects in the graphics window and display table. Interfaces are listed and selecting one of them causes a molecular-surface object to be generated (Fig. 6 ▶); the user may, for example, choose to colour this by electrostatic potential.

## 4. Publication- and presentation-quality images

### 4.1. Static views

*CCP*4*mg* offers two methods of outputting images. In the first method the pixel contents of the main OpenGL window are simply dumped to a bitmap file, while the second method is to use the included rendering program *Pixie* (http://www.renderpixie.com/), which produces high-definition output and can optionally be used to generate effects such as shadows using ray tracing or ambient occlusion. The renderer is the recommended way of producing publication-quality images. The renderer input file, in Renderman Interface Bytestream (RIB) format (https://renderman.pixar.com/products/rispec/rispec_pdf/RISpec3_2.pdf), may be saved for use with external rendering programs. Outputting the scene in a format suitable for alternative external renderers or VRML, as is performed in programs such as *Ribbons* (Carson, 1997 ▶) and *RasMol* (Sayle & Milner-White, 1995 ▶; Bernstein, 2000 ▶), is a potential future development.

Both the screen-dump and render methods allow the creation of stereo pairs and images with transparent backgrounds.

### 4.2. Movies

**Figure 7:**
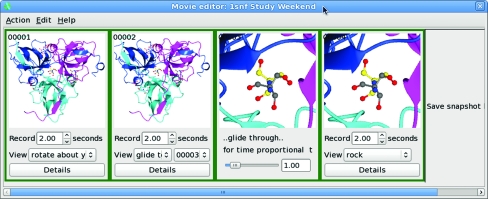
The Movie editor dialogue. The above sequence rotates an expanded view of the molecule around the *y* axis for 2 s, takes 2 s to zoom into a close-up view of a ligand and then rocks for 2 s with the ligand at the screen centre.

To generate a movie, the user only needs to set up a few key scenes that are saved as frames in the Movie editor panel (Fig. 7 ▶). The program will interpolate between these frames either to show a preview of the potential movie or to create the movie. The user can choose the number of frames inserted between each key frame to control the speed of the movie and can specify the screen size, frame rate, movie format (animated GIF or MPEG) and quality. Very high quality movies can be created using the renderer to generate each frame. While the renderer is much slower than using simple screen-pixel dumps, it offers substantially enhanced capabilities, *e.g.* much finer interpolation between different opacities of multiple objects.

*CCP*4*mg* can import the trajectory files from morphing or trajectory calculations and the Movie editor will create movies from this input. All of the usual *CCP*4*mg* display features (for example, atom selections and surfaces) can be applied to the trajectory coordinates to create sophisticated movies. Future releases will enable the calculation of morphs internally and will generate series of coordinates corresponding to a vibrational normal-mode analysis.

## 5. New features in version 2

In addition to several features described above, the move to a new programming toolkit (§[Sec sec6]6) has facilitated the development of a number of new features.

### 5.1. Toolbar and program-status history

The program’s main window contains a toolbar upon which the user is able to place an icon for any of *CCP*4*mg*’s main actions. For instance, if the user is working on creating a movie the ‘Open movie’ task may be placed onto the toolbar, obviating the need to navigate through menus every time the program is launched. The toolbar by default contains some common actions, including clearing picked atom labels and navigating backwards and forwards through the new ‘history’ feature. *CCP*4*mg* saves the program status every time a significant change is made to the scene. This status specifies which files are loaded, which atoms are selected with what styles and colours, and the camera position and orientation. The user may navigate freely backwards and forwards through this status history.

### 5.2. Inbuilt help system

The *CCP*4*mg* website (http://www.ccp4.ac.uk/MG/) has extensive HTML documentation and tutorials with help buttons throughout the program linking to the appropriate documentation. Version 2 uses the Qt Webkit tool to display help pages, replacing an earlier HTML browser that only worked in the Linux version.

### 5.3. Automatic updates

Users can opt to have the program update itself by downloading a patch with the changes to the latest version. This provides a much smaller and faster download than the whole package and is very easy to use.

Nightly (complete) builds of *CCP*4*mg* are available from http://www.ysbl.york.ac.uk/~ccp4mg/nightly/.

### 5.4. Better usage of graphics hardware

In addition to the hardware stereo capabilities in version 1, version 2 exploits the facilities of the Zalman ZM-220 series of monitors and can display side-by-side stereo pairs. Full-scene anti-aliasing is supported (and is the default) on all hardware and operating systems that support it. This includes most modern Macintosh, Microsoft Windows and Linux systems with discrete graphics adapters.

The user can easily edit the lighting of the scene in a fully flexible manner. Some simple presets are also offered. When generating publication-quality images by rendering using ray tracing to generate shadows, the program will warn if the current lighting model is unlikely to create noticeable shadows.

Full control of the depth-cue and clipping planes is provided.

### 5.5. Image rendering in batch mode

*CCP*4*mg* can run without a graphical display to render images of a scene specified as a *CCP*4*mg*-format picture-definition file, a language specification for which is available at http://www.ccp4.ac.uk/MG/ccp4mg_help2/picture_definition.html. This, for example, allows *CCP*4*mg* to be placed at the end of a computational pipeline to generate pictures of the results or the rendering facility can be called from another molecular-graphics program. This facility is also useful for testing the non-GUI parts of the program. Such testing identified some serious bottlenecks which have subsequently been eliminated.

The picture-definition language describes a single static scene comprehensively, but is not a dynamic scripting interface like those offered in *Chime* or *RasMol* (Sayle & Milner-White, 1995 ▶; Bernstein, 2000 ▶).

At present, the program will simply follow the set of commands specified on the command line and then exit. Future versions are planned to sit as a server listening for instructions.

### 5.6. Remote control

*CCP*4*mg* can be controlled to a limited extent by other programs. It is possible for other programs on either the local or a remote machine to move and orient the view and send files or request the opening of local files or to request the viewing of a whole scene. *Coot* (Emsley & Cowtan, 2004 ▶; Emsley *et al.*, 2010 ▶) is able to communicate with *CCP*4*mg* using the last of these methods. On systems with the appropriate software installed, *CCP*4*mg* may advertise its availability for such interaction using *Zeroconf* (http://www.zeroconf.org/) or an equivalent system.

The communication between programs is peformed with sockets. *CCP*4*mg* will open a socket on user request and listen for input in the background. Commands are sent as marked-up strings from client programs connected to *CCP*4*mg*’s socket. If these strings meet certain criteria then *CCP*4*mg* will interpret them as commands. For those wishing to see how this is achieved in detail, an example is distributed with the program.

### 5.7. Plug-in applications

It is possible to implement additional functionality in *CCP*4*mg* in the form of plug-in applications. Since the main portion of the program is written in Python, this is very straightforward: one simply writes a Python program con­taining the plug-in initialization function and places it in the plug-ins directory. Although the entry point of the plug-in must be a Python file, the major functionality can be written in any programming language and can access the MMDB and Clipper libraries for coordinate and experimental data handling, respectively. The plug-in interface offers the ability to load custom data types and to declare methods for display-object generation and manipulation. Several of the current *CCP*4*mg* modules are implemented as such plug-ins; for example, the *PISA* interface, the renderer, the edit-lighting GUI, automatic updates, remote control, toolbar editing and the font browser are all implemented as plug-ins. Most new features of the program will be developed in this format. Similar ideas have previously been implemented in molecular graphics such as the core/extensions architecture of *Chimera* (Petterson *et al.*, 2004 ▶).

The application-programming interface (API) still needs documentation, but the tools now exist for anyone to extend the capability of *CCP*4*mg*.

### 5.8. Single-window interface

As of version 2.4.3, *CCP*4*mg* can operate within a single window which incorporates the display table, the graphics window and the sequence viewer (Fig. 5 ▶). This is not just to create a more modern look, but also to significantly enhance the program’s usability by not having multiple windows scattered across the computer screen which can confuse the user. This is an increasingly common feature of modern computer programs such as *ICM Browser*, a molecular-graphics system with an extremely clean single-windowed interface.

## 6. Design and implementation

### 6.1. The data-handling software libraries

The core of *CCP*4*mg* is written in a combination of C++ and Python. C++ libraries handle the data and provide the computationally intensive tools for applications. Python is used to provide high-level program control and to implement the GUI; this area has undergone considerable change in version 2 and will be discussed at length in the next section.

*CCP*4*mg* uses the MMDB library developed by Eugene Krissinel for handling model data (Krissinel *et al.*, 2004 ▶). MMDB provides tools for handling PDB- and mmCIF-format files: reading and writing data, orthogonal–fractional coordinate transforms, generation of symmetry mates, editing the molecular structure plus some other higher level tasks.

Crystallographic data are handled by the Clipper libraries (Cowtan, 2003 ▶).

The Python layer of the program communicates with the C++ libraries using the *SWIG* interface generator (http://www.swig.org/).

### 6.2. Program control and the GUI

*CCP*4*mg* version 2 retains the features of version 1 and has many new features. The major change is the graphical user interface based on the Qt Application Framework (http://qt.nokia.com/). Qt creates widgets with the correct look and feel for the underlying operating system, so the same program code creates a somewhat different user interface on Windows, Mac and Linux. Qt provides an extensive collection of C++ programming tools; besides the GUI tools, *CCP*4*mg* uses Qt for displaying SVG files, spawning threads and various graphical but non-UI tasks.

Lengthy processes that would otherwise stall the GUI unacceptably (for example, downloading files and long calculations) are performed in a separate thread.

Some GUI elements use the same definitions in Python as in version 1, but are now parsed to generate Qt instead of Tk widgets; other GUI elements have been written from scratch in Qt. In both approaches, all of the final GUI definitions are Python statements; thus, a Python-to-Qt (C++) bridge is required and is currently provided by the *PyQt* (http://www.riverbankcomputing.co.uk/software/pyqt/intro) bindings package. The *PySide* bindings developed by Nokia will be considered as a replacement as soon as a stable version is released.

## 7. Summary and future plans

We have created a much improved version of the *CCP*4 molecular-graphics program: *CCP*4*mg* now contains a com­prehensive set of tools for both automatic and very specific user-defined atom selections, the output image quality has been improved by the incorporation of a rendering module, interface and multimer analysis is possible using *PISA* and work has begun on sequence alignment and exploration features, and communication with the *Coot* model-building program has improved and will continue to do so.

Future releases will include enhancements to the sequence viewer, including the ability to specify weighting to parts of the alignment, gap penalties *etc*. and the ability to change the style of the structure displayed when the user highlights residues in the sequence. Colouring the three-dimensional structure by sequence conservation will be enabled. Work on normal-mode visualization is in progress: a normal-mode program exists, a GUI needs to be written and we plan to visualize by creating animations for each mode and providing a ‘porcupine’ static representation mode. Polysaccharide cartoons and multiple bond displays (using information from the CCP4 monomer library) will be implemented.
